# Training-Associated Superior Visuomotor Integration Performance in Elite Badminton Players after Adjusting for Cardiovascular Fitness

**DOI:** 10.3390/ijerph19010468

**Published:** 2022-01-01

**Authors:** Yi-Liang Chen, Jen-Hao Hsu, Dana Hsia-Ling Tai, Zai-Fu Yao

**Affiliations:** 1 Graduate Institute of Sports Training, University of Taipei, Taipei City 111036, Taiwan; yiliang@utaipei.edu.tw (Y.-L.C.); jenhao@mx.nthu.edu.tw (J.-H.H.); tscsa@utaipei.edu.tw (D.H.-L.T.); 2 Physical Education Office, National Tsing Hua University, Taipei City 300044, Taiwan; 3 Department of Physical Education, University of Taipei, Taipei City 111036, Taiwan

**Keywords:** badminton, integration, anticipation, cognition

## Abstract

Badminton is recognized as the fastest racket sport in the world based on the speed of the birdie which can travel up to 426 km per hour. On the badminton court, players are not only required to track the moving badminton birdie (visual tracking and information integration) but also must anticipate the exact timing to hit it back (temporal estimation). However, the association of training experience related to visuomotor integration or temporal prediction ability remains unclear. In this study, we tested this hypothesis by examining the association between training experience and visuomotor performances after adjusting for age, education, and cardiovascular fitness levels. Twenty-eight professional badminton players were asked to perform a compensatory tracking task and a time/movement estimation task for measuring visuomotor integration and temporal prediction, respectively. Correlation analysis revealed a strong association between training experience and performance on visuomotor integration, indicating badminton training may be promoted to develop visuomotor integration ability. Furthermore, the regression model suggests training experience explains 32% of visuomotor integration performances. These behavioral findings suggest badminton training may facilitate the perceptual–cognitive performance related to visuomotor integration. Our findings highlight the potential training in visuomotor integration may apply to eye–hand coordination performance in badminton sport.

## 1. Introduction

Badminton is recognized as the fastest racket sport in the world based on the speed of the birdie [1] which can travel as fast as 426 km per hour, as booked in the Guinness World Record. Players are required to have quick reflexes and good stamina. Badminton players typically use the racket as a mediator to utilize more power from it. Therefore, an excellent badminton player must produce the intermediary object to respond to the opponent. On the badminton court, an elite badminton player is not only able to track the moving badminton birdie but also must anticipate the exact timing for hitting the target. These skills can be generally involved in a process of visuomotor integration and a process of temporal prediction for success in badminton performance. However, whether visuomotor integration and temporal prediction are associated with sports-specific training experience in badminton sports remain unclear.

Visuomotor integration is playing an important role in the coordination between visually perceived external information and appropriate motor response to react. Efficient visuomotor integration depends on integrated multisensory processing by incorporating both the visual and motor systems of the brain. Visual-motor skills, known as visual-motor integration are the ability to interpret visual information and respond with a motor action. These skills include eye–body coordination, sports, and any activity that involves physical activity. For instance, an athlete sees a baseball approaching while an athlete responds by moving their hands to seize the baseball. Studies report that visuomotor skills are related to eye–hand coordination skills [2,3,4]. Eye–hand coordination is the developmental skill of the central nervous system to incorporate the information received from the eyes to control, guide, and direct the limbs to react for performing a given task such as catching a ball [5]. So far, only a handful of studies have investigated the eye–hand coordination of badminton players [6,7,8]. Dane, Hazar, and Tan [6] suggested that badminton training was associated with superior eye–hand visual reaction time and visuospatial intelligence. Dube, Mungal, and Kulkarni [8] evident that the visual reaction time in badminton players was faster than sedentary controls. Specifically, for instance, if visually introduced information is not perceived correctly, the muscles will have received incorrect messages leading to inappropriate motor responses. Moreover, a study [9] found that children who have deficits in visuomotor integration may express difficulties with participating in sports that involved eye–hand coordination skills, eye–foot coordination skills, and coordination of both sides of the body. More specifically, they were unable to complete motor skills such as daily living activities, copying visual information, body awareness, reading, lining up math problems, handwriting, geometry, and speed of complete motor tasks. On the contrary, visual gaze behavioral training [10] and physical exercise training [11] can improve visuomotor integration in children with developmental coordination disorder. Despite the importance of visuomotor integration in the execution motor response, how this ability is related to specific sports training remains unclear. No prior study has linked visuomotor integration in sports expertise. Hence, our study aimed to investigate if training experience is promoted to develop visuomotor integration.

Real-time recognition of the development of dangerous circumstances is of pivotal importance for the rapid execution of countermeasures. For instance, a skill that quickly and correctly predicts the time-to-contact of moving objects that become significantly concealed is advantageous in daily living activities and could be of utmost critical in fast-ball sports. Only one study [12] investigated temporal prediction ability in sports expertise (i.e., rugby players and cricket players). Their results suggest that there was no difference between sporting groups and controls on temporal prediction ability but there were significant independent effects on performance variables including target speed, destination speed, and occlusion period. However, whether these findings are associated with sports-specific training is still unclear. Despite research on the mechanism of anticipation skills in sports being lacking, research to enhance anticipation skills in sports to help better athletic performances on the field are fruitful. For example, a study [13] showed tennis players who received cognitive-perceptual training improved their performance on both laboratory- and field-based tests of anticipation as compared to both matched placebo and control groups. Nonetheless, whether superior temporal prediction is facilitated by sport-specific training experience remains inconclusive.

Despite the possible link between training-associated visuomotor performance changes that may manifest in the elite athlete, possible confounds of physical exercise-induced cardiovascular levels on task performance should be considered. For example, a previous study [14] examined whether cardiovascular fitness and cognitive task type moderate the relationship between acute exercise and cognition, results showing that healthy adult participants of all cardiovascular fitness levels improved in performance of various task performances after a single bout of exercise. Moreover, a larger-scale study [15] with over one million young adults participating showed that cardiovascular fitness changes between ages 15 and 18 years old predicted cognitive performance at the age of 18. Importantly, these findings reported the facilitated effects of exercise-induced cardiovascular fitness levels on cognitive performance. Thus, to test if training-associated experiences are related to visuomotor performances, it is important to rule out the possible confounds of cardiovascular fitness levels on visuomotor performances.

Overall, the goal of this study was to determine if training experience is associated with visuomotor integration or temporal prediction in elite badminton players. We tested if training-dependent visuomotor abilities change associated with sports-specific training experience after adjusting for possible confounds. Specifically, elite badminton players were asked to perform a compensatory tracking task and a time/movement estimation task for measuring visuomotor integration and temporal prediction, respectively. The association between training experience and perceptual–cognitive performance was investigated.

## 2. Materials and Methods

### 2.1. Participants

A total of 28 elite badminton players (aged 20–30 years old; 14 males and 14 females) were voluntarily recruited to participate in this study. These players played in professional badminton clubs in the country. Most of them were qualified for representing Chinese Taipei at international sports competitions (e.g., summer Olympics). These players were all listed in the Badminton World Federation (BWF) rankings. All players gave informed consent before the experiment. Basic demographic information (age, gender, anthropometric measurements (e.g., height and weight)), education levels, and fitness levels were collected. All adult players (age >20 years old) were given written consent to inform the experimental procedures before their participation. All participants were recruited by the advertisement flyer that was dispatched around the campus. The University of Taipei provides distinct academic recognition for those who are concurrent top professional athletes in Taiwan, which makes it less painful to have these elite athletes participate. They were instructed to perform a series of computer tastings for reward as one research participation credit for their chosen statistical and experimental design course. After giving informed consent, players were screened following the guideline by the American College of Sports Medicine (ACSM) Guidelines for Exercise Testing and Prescription, 10th edition to ensure that all players were rated as a low risk before exercise testing [16]. Players who were not at low risk did not participate in this study [17]. The ethics of experimental procedures were approved by the ethics committee on human experimentation by the Institutional Review Board at the UT.

### 2.2. Physical Fitness Assessment

Physiological fitness levels including the maximum rate of oxygen consumption (VO^2^ max) and maximum heart rate (HR max) were collected by Treadmill Test with H/P Cosmos Pulsar, H/P/COSMOS 3P 4.0^®^, Nussdorf-Traunstein, Germany. The VO^2^ max was measured using a cardiopulmonary diagnostic system (Cosmed Quark CPET system; Rome, Italy) based on the Bruce Treadmill Protocol [18,19]. For a Bruce Protocol, the test score is the time taken every three minutes while the speed and incline of the treadmill are increased. The treadmill is started at 2.74 km per hour and an inclined gradient of 10% After 3 min incline of the treadmill is increased by 2%, and the speed increases. The test should be stopped when the subject cannot continue due to physical or mental reasons.

### 2.3. Compensatory Tracking Task: Visuomotor Integration

The purpose of this task was to utilize an intermediary object (i.e., mouse) for controlling to maintain a random-drifting trackball on the mark of the circle for 20 s. Participants were asked to continuously monitor and adjust the cursor to keep it within the gray circle. The paradigm [20] was originally designed by Makeig and Jolley (1995) and was modified in a computer software program [21] by Mueller and Piper (2014). In this task, participants were asked to utilize visual information to guide motor planning, execution, and modification, that involves accuracy demands, small directional changes in the trajectories to incorporate instant information that was updated which was taken as evidence of feedback-based error corrections to add to the literature regarding visuomotor integration [22]. A total of 24 trials were completed. The overall performance was to integrate distance implied by the x and y direction offsets (in pixels from the center) of the target over time and use that as a deviation score. The performance was then calculated as the summing deviation score and the time delta as integration abilities. The higher value indicates better performance to keep a randomly drifting ball ‘on target’.

### 2.4. Time/Movement Estimation Task: Temporal Prediction

This task was originally designed by Jerison et al. (1957) [23] and used as a modified version [24] of the time/movement estimation task by Webb et al. (2013) [24], in which a moving object vanishes behind a block, and the participant must estimate when is the timing for the moving objects to pass through the target. Time perception is fundamental for human experience ranging from daily activities to sports performances. This task was described in the United-Tri services Performance Assessment Battery. Performance is a proportionality score that divides the difference between correct and response by the correct time so that a value close to 0 indicates better prediction accuracy.

### 2.5. Experimental Apparatus

All behavioral testing was performed on a laptop (ACER Core™ i5 TravelMate P245) with 14” displays, and the participants made responses via the keyboard and the mouse. The tasks were presented with an open-source software program on the Windows operating system, developed by Mueller & Piper (2014), namely the Psychology Experiment Building Language (PEBL) [21].

### 2.6. Experimental Procedures

Consent forms were prepared and demographic information was documented such as age, height, body weight, and gender of participants. Warm-up exercises were performed before testing started. All participants were firstly tested with Bruce protocol [25] for a physical fitness assessment (i.e., VO^2^ max). After resting until the heart rate was back to normal (10–15 min), all participants were asked to be seated in a laboratory chamber to perform a behavioral experiment with a laptop setup. Behavioral experiments were administered in the following order for all participants: (1) compensatory tracking task; (2) time/movement estimation task; these tasks fell into two major cognitive categories: (a) visuomotor integration, (b) temporal prediction. To reduce potential fatigue effects, there was a one-minute break between consecutive tests. The entire experimental session lasted for 1 h and 30 min to complete.

### 2.7. Statistical Analysis

To examine if training experience is associated with visuomotor integration or temporal prediction in elite badminton players, Pearson’s r correlation analysis among behavioral variables was conducted. Pearson coefficients and *p*-value were used to measure the linear bivariate correlation. The correlation analysis examines whether the badminton training experience is associated with the ability to track the moving badminton birdie (visual tracking and information integration) or able to anticipate the exact timing to hit it back (temporal estimation) adjusting for age, education, and cardiovascular fitness levels. Linear regression models were next used to explain the relationship between criterion variables and predictor variables. Moreover, an independent *t*-test was conducted to examine if gender differences exist. R-squared (R2) is a measure for a regression model that represents the proportion of the variance for a dependent variable explained by an independent variable [26]. The significance level was set at alpha = 0.05. All analyses were performed after adjusting the age, years of education, BMI, HR max, and VO^2^ max as covariates. All analyses were conducted using IBM SPSS Statistics (Version 24. IBM Corp. in Armonk, NY, USA). Moreover, Bayesian statistics on the correlation model were performed in R (The R Project for Statistical Computing, version 4.0.5) using BayesMed toolboxes to test for the association between visuomotor integration or temporal prediction and years of training experience while controlling for age, education, and cardiovascular fitness levels as a covariate of no interest. Furthermore, Bayesian statistics were performed on the linear regression model to examine the links between years of training experience in badminton and cardiovascular fitness level. Specifically, we performed Bayesian hypothesis testing via Bayes factors that calculated the ratio of the marginal likelihoods for the hypothesis-specific model. Bayes Factors (BF) [27] were calculated to assess the presence of a correlation or regression and can be interpreted as proportional evidence for the presence or absence of an effect. For instance, a BF10 of 5 indicates that the data are 5 times more likely to occur under the alternative hypothesis than under the null hypothesis [28,29].

## 3. Results

### 3.1. Demographic Information

Participants’ demographic information is reported in Table 1. The average age across participants who were first-division national players was 21.35 ± 2.65 (Mean ± SD). The average education level was 16.6 ± 1.6 years. The average years of training experience were 12.2 ± 3.75 years. Specifically, the average height for the male player was 173.4 ± 6.73 cm, whereas the average height for a female player was 164.5 ± 4.4 cm. Moreover, the average weight for the male player was 67.9 ± 7.5 kg whereas the average weight for a female player was 60.4 ± 5.5 kg. The average body mass index (BMI) for the male player was 22.5 ± 1.9 kg/m^2^, whereas the average BMI for the female player was 22.3 ± 1.5 kg/m^2^. The average VO^2^ max for the male player was 60.38 ± 8.9 mL/kg/min, whereas the average VO^2^ max for the female player was 46.81 ± 9.6 mL/kg/min. The average HR max for the male player was 187.3 ± 11.3 *bpm*, whereas the average HR max for the female player was 180.1 ± 14.1 *bpm.*

### 3.2. Behavioral Performances

The average performance scores of a compensatory tracking task across participants were 8.891e − 4 ± 0.007. The average reaction time (RT) of a time/movement estimation task across participants was 5918.986 ± 622.877 milliseconds (msec). The average accuracy of a time/movement estimation task across participants was 0.056 ± 0.016 msec. No significant gender differences among compensatory tracking task performances (paired t(26) = 0.086, *p* = 0.932) and time/movement estimation task performances on RT (paired t(26) = −1.068, *p* = 0.295) and the task accuracy (paired t(26) = 1.488, *p* = 0.16) were observed.

### 3.3. Correlation Analysis

**Figure 1:**
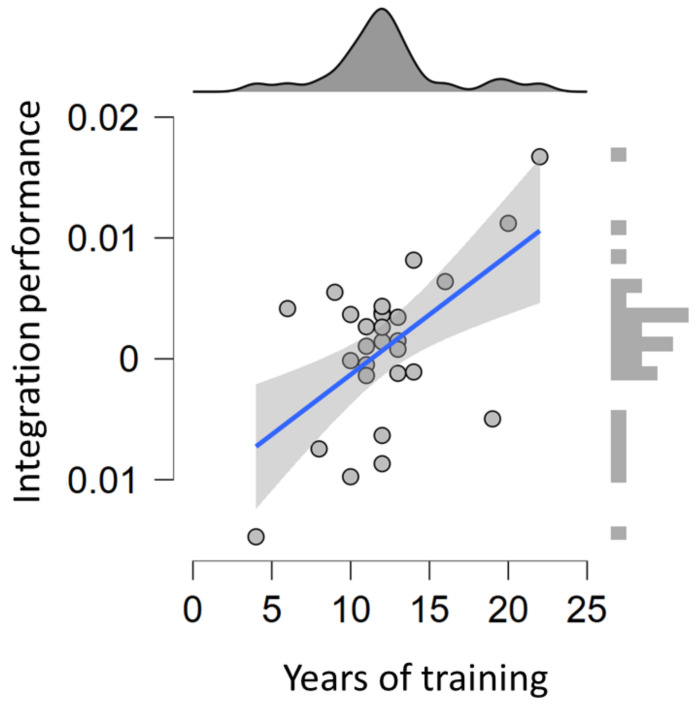
Associations between years of training and compensatory tracking task performance.

**Figure 2:**
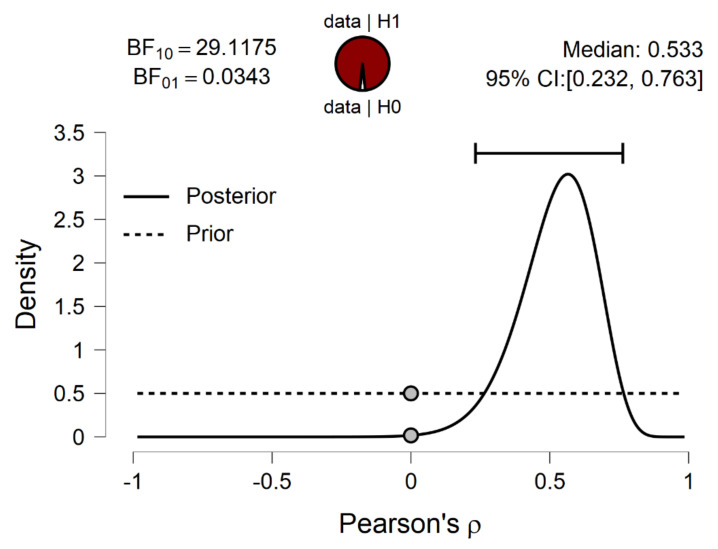
Prior and posterior of Bayesian correlation model.

**Figure 3:**
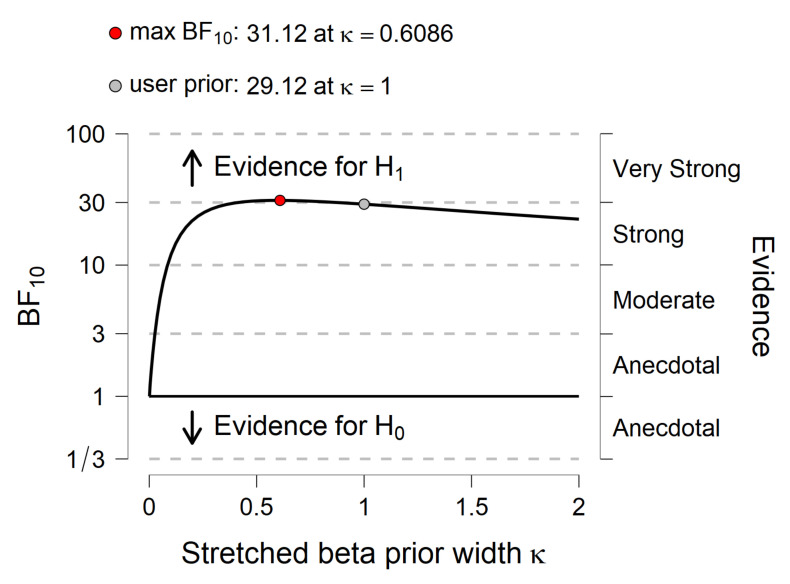
Bayes Factor (BF) robustness check.

The association between years of training and behavioral performance measures on average accuracy of the time/movement estimation was examined. Results showed that a strong positive association between compensatory tracking task performance and years of training experience was observed (Pearson’s r = 0.573, *p* = 0.001, BF_10_ = 29.117; Figure 1, Figure 2 and Figure 3). No significant correlation between years of training and time/movement estimation accuracy was observed (Pearson’s r = 0.128, *p* = 0.516). Further linear regression model between compensatory tracking task performance and years of the training experience reported R-squared (R^2^) equal to 0.328, BF_10_ = 1.00 (Figure 1) for testing the predictive value of years of training experience in compensatory tracking task performance.

## 4. Discussion

This study aimed to determine if sport-specific training is associated with the improvement of visuomotor integration or temporal prediction performance in elite badminton players. Results suggest that years of sports training experience are associated with better visuomotor integration in elite badminton players, indicating badminton sports training may promote sports-related skills in eye–hand coordination instead of movement anticipation.

The importance of hand–eye coordination cannot be overemphasized in modern sports training [30,31,32]. Simply put, it is how well eyes are able to control the movement of hands, for instance, eyes send signals to the brain, which directs the movement of the hands. Excellent visuomotor integration depends on incorporated processing by both visual and motor systems of the brain [33,34]. Individuals with good hand–eye coordination are able to process external information quickly and effectively [35]. The hands and eyes must coordinate together to accomplish a skill. How well that skill is achieved depends on the strength of the visual connection from the eyes to the brain to the hands. Visual–motor skills as part of visuomotor integration are the ability to interpret visually perceived external information and react with a motor response. These processes result in them having a better reaction time and enhanced athleticism, a distinguishing factor between a great athlete and a not-so-great athlete. Our findings suggest that practice (i.e., badminton training experiences) can improve that connection for elite badminton players.

Studies have shown that sport-specific training, such as eye–hand coordination drill training, is associated with actual athletic performance. For example, a previous study [36] investigated visual–motor reaction time and its relation to the baseball performance on batting in elite baseball players. Results showed a significant association between the visual–motor reaction time ability and batting performance. This finding demonstrates the importance of visuomotor performance in relation to actual game performance. These abilities (i.e., visual–motor reaction time) represent the integration of visual information, perceptual decisions, and motor movements to achieve a complex task. These interpretations may represent the possible role of visuomotor integration in the performance of actual athletic fields. However, evidence remains unclear if these abilities are related to sports-specific training. To fill this gap, our findings linked the sports-specific training associated with visuomotor integration ability in elite badminton players.

Anticipating the timing of coming objects is a necessary precursor to preparing actions and allocating resources to sensory–motor processing [37]. Useful predictions typically do not arise from the beginning [38]. Timepass almost affects every aspect of human behavior. We depend on the sense of elapsed time to plan actions and anticipate salient environmental events to guide behavior [37]. For instance, it takes time to experience and practice for a baseball player to precisely anticipate a particular type of pitch, predict the trajectory of the ball by combining top-down information about a particular pitch with the information about the bottom-up perception of the rotation/speed of the ball, and make a decision regarding when and how to swing at it [39]. The description of the sporting events is all about temporal prediction. This ability to utilize the stored knowledge and learned modes of behavior reduces the need to consider a large number of potential causes or courses of action, which enables quicker interpretation of endogenous and exogenous events, and faster, more precise, and less effortful responses [39]. In our study, results found that training-associated temporal prediction errors may not increase or reduce with training experiences. This demonstrates that prediction accuracy may not be affected by training as players have exhibited superior performance on perceptual–cognitive abilities, but action anticipation excellence in sports expertise may be associated with the fine-tuning of specific anticipatory ‘resonance’ mechanisms [40] that endow elite athletes’ brains with the ability to movement estimation which may explain why temporal prediction did not covariate with training experience. Another possible explanation may be due to the experimental setup not being conducted under a sports-specific context [41,42], which may explain why we did not observe in a traditional laboratory setting tasks where the testing environment is stripped of the sport-specific context.

The idea of perceptual–cognitive training has been recently advocated [43,44,45]. For example, a previous study [46] examined whether sports-related perceptual–cognitive training can be observed in the top competitive sports expertise. They trained a large sample of soccer players on a complex dynamic visual–motor required task. Their results suggest that professionals as a group dramatically differ from high-level amateur athletes, who dramatically differ from non-athlete university students in their capacity to learn such stimuli. These findings resonate with a recent review [43] that future perceptual–cognitive training in athletes may help to process stimuli faster which may be promising for future sports training. Nonetheless, little is known about training-associated perceptual–cognitive abilities that are related to the sports-specific context (i.e., badminton sports). Our findings added to evidence that training experience may facilitate certain perceptual–cognitive abilities (i.e., visuomotor integration) in a sport-specific context (i.e., badminton players), overlapping skill sets between perceptual–cognitive abilities (i.e., visuomotor integration) and sports training drills (i.e., eye–hand coordination) are subject to protracted badminton training.

Moreover, Voss et al. (2010) recommended that future studies on sports and cognition should be adjusted for gender variables and use a diverse range of sport types with different levels of expertise. The authors suggest that sports types would be a potential moderator variable that makes use of different mental demands and unique athletic experience-dependent plastic changes in sport expertise [47]. Our findings added to these notions that overall matched numbers of badminton players showed no difference in both task performances. Some of the gender differences in neuropsychological performances have been attributed to culture and education [48]. For example, studies have shown sports-related training/practice can reduce the gender performance gap of spatial abilities [49,50]. In line with these findings, it might suggest that gender differences of sports expertise on behavioral performance related to perceptual–cognitive abilities are minimized if male and female athletes are given equal opportunities for similar experiences, learning, and training [51]. This notion would explain why gender differences are typically observed in healthy controls, not in athletes [52].

Despite the findings presented in the current study, we observed the sport-specific training experience on visuomotor integration in elite badminton players. Few limitations have to be reflected in future studies. Despite our utmost effort to recruit quite a large numbers of elite badminton players to participate, the sample size is still limited. Due to the nature of the training schedule in the top sports expertise, elite badminton players in this study are still difficult to access, and hence the sample size is unavoidably small. Furthermore, the cycle of training differing in badminton players may also add to the confounding effects for presenting the results. For instance, few badminton players were preparing for a global competition in months, whereas others had just finished their national competition in previous days. Furthermore, the participants in this study played at an elite level, most of them competed at international events and were ranked top by the Badminton World Federation (BWF). However, some concerns regarding the definition of sports expertise level are still unsettled [53]. This confounding may increase performance variances and individual differences. Future studies should take these notes into account for designing experiments to study the perceptual–cognitive abilities in sports expertise. Moreover, in this study, we collected the information through a self-report measurement, which may be biased when they report on their own experiences [54]. For example, athletes are either consciously or unconsciously influenced by social desirability. Therefore, they are more likely to report longer training experiences that are considered to be socially preferred. Future studies should acquire quantity data from their past sports competition experience to determine a more accurate measurement.

To mitigate possible confounding effects of cardiovascular fitness levels on these behavioral performances, we also collected physiological data in elite badminton players. The purpose of cardiovascular fitness inclusion is to determine if the association between the sport-specific training experience and visuomotor integration in elite badminton players was affected by the individual’s aerobic capacity. Previous studies have found that increased cardiovascular fitness seems to improve certain domains of cognitive performance, with the underlying assumption that aerobic capacity facilitates neuroplasticity [55]. Our findings suggest that the superior visuomotor integration in elite badminton players is independent of cardiovascular fitness levels, meaning that observed sports-specific training on visuomotor integration abilities is possibly experienced-driven and related. These findings provide empirical evidence to support the notion of cognitive training in a sport that may be enhancing cognitive skills through repetitive sporting training leading to improved performance in sport.

## 5. Conclusions

This study provides the first attempt to investigate the association between training-associated perceptual–cognitive abilities pertaining to badminton sports. Specifically, we hypothesized that an elite badminton player is not only able to track the moving badminton birdie but also must anticipate the exact timing for hitting the target. These skills can be generally categorized as visuomotor integration and temporal prediction for success in badminton performance. Our behavioral findings suggest years of sports training experience are associated with superior visuomotor integration in elite badminton players, indicating badminton sports training may promote sports-related skills in eye–hand coordination instead of movement anticipation. The findings can be explained by sports-specific training in badminton players and their improvements in eye–hand coordination skills. This study highlights the potential application of perceptual–cognitive training that may help to develop well-rounded athletic performance.

## Figures and Tables

**Table 1:** Demographic information of participants in this study.

	Elite Badminton Players
N	28
Age	21.35 ± 2.65
Education	16.6 ± 1.6
Training (years)	12.214 ± 3.755
Height	168.8 ± 6.9
Weight	64.1 ± 7.5
BMI	22.4 ± 1.75
VO^2^ max (mL/kg/min)	53.3 ± 10.7
HR max (*bpm*)	183.8 ± 14.1

N = number of participants; Training = years of training experience; BMI = body mass index; VO^2^ max = maximum rate of oxygen consumption; HR max = maximum heart rate; kg = kilogram; min = minute; ml = milliliters of oxygen; *bpm* = beats per minute.

## Data Availability

The data presented in this study are available on request from the corresponding author. The data are not publicly available due to containing information that could compromise the privacy of research participants (i.e., elite athletes).
